# The Magnitude of Peripheral Muscle Fatigue Induced by High and Low Intensity Single-Joint Exercise Does Not Lead to Central Motor Output Reductions in Resistance Trained Men

**DOI:** 10.1371/journal.pone.0140108

**Published:** 2015-10-06

**Authors:** Paul W. M. Marshall, Harrison T. Finn, Jason C. Siegler

**Affiliations:** 1 Human Performance Laboratory, School of Science and Health, University of Western Sydney, Sydney, Australia; 2 Neuroscience Research Australia (NeuRA), Sydney, Australia; UFMG, BRAZIL

## Abstract

**Purpose:**

To examine quadriceps muscle fatigue and central motor output during fatiguing single joint exercise at 40% and 80% maximal torque output in resistance trained men.

**Method:**

Ten resistance trained men performed fatiguing isometric knee extensor exercise at 40% and 80% of maximal torque output. Maximal torque, rate of torque development, and measures of central motor output and peripheral muscle fatigue were recorded at two matched volumes of exercise, and after a final contraction performed to exhaustion. Central motor output was quantified from changes in voluntary activation, normalized surface electromyograms (EMG), and V-waves. Quadriceps muscle fatigue was assessed from changes in the size and shape of the resting potentiated twitch (Q._pot.tw_). Central motor output during the exercise protocols was estimated from EMG and interpolated twitches applied during the task (VA_sub_).

**Results:**

Greater reductions in maximal torque and rate of torque development were observed during the 40% protocol (p<0.05). Maximal central motor output did not change for either protocol. For the 40% protocol reductions from pre-exercise in rate and amplitude variables calculated from the Q._pot.tw_ between 66.2 to 70.8% (p<0.001) exceeded those observed during the 80% protocol (p<0.01). V-waves only declined during the 80% protocol between 56.8 ± 35.8% to 53.6 ± 37.4% (p<0.05). At the end of the final 80% contraction VA_sub_ had increased from 91.2 ± 6.2% to 94.9 ± 4.7% (p = 0.005), but a greater increase was observed during the 40% contraction where VA_sub_ had increased from 67.1 ± 6.1% to 88.9 ± 9.6% (p<0.001).

**Conclusion:**

Maximal central motor output in resistance trained men is well preserved despite varying levels of peripheral muscle fatigue. Upregulated central motor output during the 40% contraction protocol appeared to elicit greater peripheral fatigue. V-waves declines during the 80% protocol suggest intensity dependent modulation of the Ia afferent pathway.

## Introduction

Mechanisms of muscular fatigue are generally considered from peripheral factors associated with maintaining muscle contraction (e.g. blood flow, oxygen delivery, contraction efficiency), and factors associated with maintaining central motor output to the muscle from the nervous system (e.g. cortical and motoneuron output) [1,2]. In the intact muscle of the exercising human, the manipulation of intensity and total volume of work is thought to influence the relationship between peripheral muscle fatigue and central motor output [3]. However, research investigating whether or not the relative contraction intensity (e.g. high or low) of an exercise protocol elicits a differential effect on this relationship is contradictory [3–6]. Moreover all of these studies tested relatively untrained healthy or university student populations, limiting the generalizability to resistance trained populations where fatiguing exercise at high and low contraction intensities are routinely performed. This demographic is of particular interest for examination of, and comparison between, different contraction intensities during fatiguing exercise considering the chronic adaptation unique to the nervous system of resistance trained individuals.

Resistance trained individuals manifest several neural adaptations, specifically reported as increased supraspinal drive [7–9], greater peripheral twitch responses to cortical stimulation as well as decreased responsiveness to cortical and peripheral nerve stimulation during performance of sub-maximal contractions [10–12]. Combined, these findings suggest a greater ability of trained individuals to voluntarily recruit the available motor unit pool, and an increased input-output response at the level of the α-motoneurone [10–12]. For resistance trained individuals, fatiguing single joint exercise at high and low contraction intensities is a typical example of a real-world training session. Investigating the interaction between peripheral muscle fatigue and central motor output in a resistance trained population during such exercise may help clarify the discrepancy between studies previously investigating the influence of contraction intensity [3–6].

In single joint knee extensor exercise performed in healthy young untrained populations it appears that peripheral muscle fatigue is not regulated by measurable declines in central motor output [13,14]. For example, despite observing greater reductions in peripheral fatigue (e.g. a decline in the quadriceps potentiated twitch (Q._pot.tw_)) when inspired gas fractions of O_2_ (F_I_O_2_) were reduced to 10% during high intensity knee extensor exercise, Christian and colleagues reported no influence of F_I_O_2_ on the decline in central motor output [13]. Similar findings were observed in a study comparing single and double leg cycle ergometer based knee extensor exercise [14]. While greater Q._pot.tw_ reductions were observed in the single leg condition, declines in central motor output were not different between either exercise condition [14]. Combined, these findings suggest that in single joint exercise the central nervous system is capable of tolerating varying levels of peripheral muscle fatigue without manifesting a de-recruitment often thought to pre-empt deleterious levels of peripheral fatigue in whole body exercise models [15]. Considering the extent of neural adaptation evident in resistance trained individuals, it is unclear if any changes in central motor output will be observed during single joint fatiguing exercise regardless of the exercise intensity or extent of peripheral muscle fatigue.

Changes in central motor output during single joint fatiguing exercise can be examined from measures such as voluntary activation (VA) and V-waves. VA is calculated from the relative change in the size of an electrically evoked twitch superimposed on a maximal contraction with the size of the Q._pot.tw_ [16]. The presence of a superimposed twitch during maximal contraction suggests sub-optimal output from the α-motoneurone [2]. Thus an increase in the superimposed twitch during a fatiguing protocol is evidence of reduced central motor output. The V-wave is a variant of the H-reflex elicited during maximal contraction because the collision between high levels of descending drive and the antidromic signal elicited from peripheral nerve stimulation allows the Ia afferent volley to propagate to the muscle [17]. Thus the V-wave is thought to reflect the level of supraspinal drive [18], although pre- and post-synaptic changes may also mediate this evoked potential [8,19]. Declines in soleus V-waves following fatiguing calf exercise have been observed concomitant to reduced VA, thus supporting the use of V-waves as an additional measure of fatigue induced changes in central motor output [20]. To our knowledge no study has examined quadriceps V-waves following fatiguing single joint exercise.

Examining changes in the size and shape of the Q._pot.tw_ provides measures of peripheral muscle fatigue that may discriminate between high and low intensity exercise in resistance trained individuals. High-intensity fatiguing exercise is more likely to achieve recruitment and thus fatigue of a high proportion of, if not all, the type II muscle fibers [21,22]. Type II fibers, and in-vivo measures from muscles with higher type II fiber content, exhibit faster temporal twitch characteristics (e.g. time to peak twitch, rate of twitch increase, ½ relaxation time) than type I fibers [23,24]. Therefore a high-intensity fatiguing protocol may be more likely to exhibit changes in temporal measures of the evoked Q._pot.tw._ Alternatively a fatiguing low intensity protocol, especially if performed to the point where the contraction intensity can no longer be maintained and therefore a greater exercise volume is accrued, may facilitate progressive recruitment of higher threshold motor units [25]. In this instance, peripheral fatigue may be exacerbated more from low intensity exercise where substantial fatigue of both the type I and II motor unit pools is likely to occur.

We designed this study to address gaps in the understanding of peripheral muscle fatigue and central motor output during high and low contraction intensity single joint exercise in resistance trained individuals. We hypothesized that 1) rate based measures of the Q._pot.tw_ would decline earlier and to a greater extent in the high intensity protocol, 2) central motor output during maximal voluntary contractions would decline similarly during high and low intensity exercise protocols, and 3) amplitude based measures of the Q._pot.tw_ would be reduced to a greater extent after the low intensity protocol was performed to exhaustion.

## Materials and Methods

### Subjects

Ten resistance trained men volunteered to participate in the study (age, 26 ± 4.5 years; height, 178 ± 5.2 cm; body mass, 86 ± 9.7 kg; training experience, 5.7 ± 3.5 years; mean ± SD). All participants were required to have regularly (at least 4 times per week) performed resistance exercise for the upper and lower body for at least the last 24-months. Exclusion criteria were any reporting of taking performance enhancing substances as per the World Anti-Doping agency’s 2012 list, any recent history of lower limb injury (last 3-months), or any known metabolic or neuromuscular disease. Written informed consent was obtained from each participant. All procedures were approved by the University of Western Sydney Human Research Ethics Committee. The experimental protocols conformed to the Declaration of Helsinki.

### Experimental design

Participants attended the laboratory on three occasions. At a preliminary visit, participants were thoroughly familiarized with procedures used to assess knee extensor maximal voluntary isometric torque and perform the exercise task (KinCom 125 dynamometer, Version 5.32, Chattanooga, USA), and peripheral muscle fatigue and central motor output based on the interpolated twitch technique. On two separate days in a random order, with five to seven days between sessions, participants performed the exercise protocol at an intensity of either 80% or 40% of their maximal voluntary isometric torque (MVT). MVT was measured at the start of and throughout each experimental session.

### Exercise protocol

The exercise protocol was preceded by a warm-up series of six 3-5s sub-maximal contractions that increased in self-perceived intensity of the participant from 2 x 25%, 2 x 50%, and 2 x 75%. Participants were then required to perform four maximal torque efforts in a random order separated by 1-minute, with two efforts involving interpolation of a supramaximal electrical stimulus to the femoral nerve and two efforts with no stimulation. MVT was determined from the trial with the highest torque, and the torque output for the exercise protocol was calculated. Torque output was continuously displayed to participants on a 25” LCD monitor (LG™, Australia), with horizontal lines placed ± 2.5% around the required contraction intensity. Throughout the exercise trial participants were required to maintain torque output within these limits. Strong verbal encouragement was always provided.

**Fig 1:**
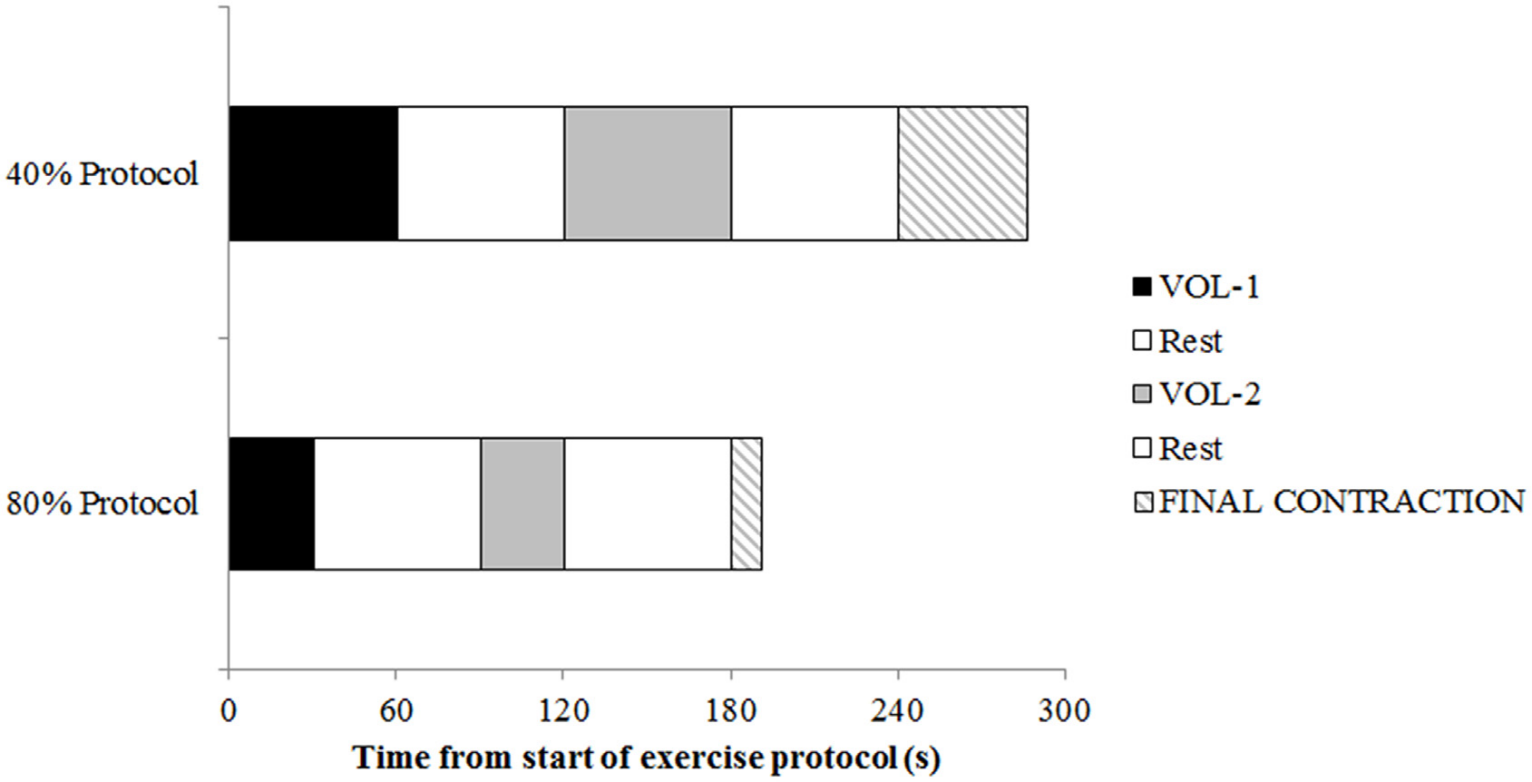
Overview of the experimental protocol used in this study. Maximal voluntary torque was measured pre-exercise, after VOL–1, VOL–2, and the end of the final contraction performed to exhaustion.

The 80% exercise protocol required 60s of total contraction time. This was divided into two 30s contractions, with 1-min rest (Fig 1). Pilot work found that 30s was the highest multiple of 10 that could be sustained by similarly trained participants without failure for an initial contraction. 1-min rest was determined as the minimum rest period where participants were able to recover sufficiently to reach the 80% torque level again. MVT was measured immediately after 30s and 60s. The 40% protocol required 120s of total contraction time. To allow comparisons between-protocols after matched exercise volumes, MVT was measured at 60s (VOL–1) and 120s (VOL–2) during the 40% protocol, with 1-min rest provided after each maximal effort.

If the required torque could not be maintained in either protocol (torque output below lower limit for 2s), the contraction was immediately stopped and 1-minute rest was provided before continuing.

Following 60s at 80% and 120s at 40% 1-min rest was provided, and participants were then required to perform a final contraction at the required intensity until they could no longer maintain the required contraction intensity (Table 1). MVT was measured after the final point of exhaustion. Femoral nerve stimulation was applied during and 2-3s after all MVTs to examine central motor output and peripheral muscle fatigue.

**Table 1:** Pre-exercise muscle function and performance data during the 40% and 80% protocols. All participants completed the VOL–1 workload without stopping. 3 out of 10 participants completed the second 30s contraction at 80% without stopping (VOL–1 to VOL–2). 6 out of 10 participants completed the second 60s contraction at 40% (VOL–1 to VOL–2) without stopping.

	40% Protocol	80% Protocol
MVT (Nm)	280.1 ± 48.3	258.9 ± 53.0
VA (%)	90.1 ± 7.7	90.8 ± 9.9
VL M-wave (mV)	12.3 ± 5.3	12.1 ± 5.6
VM M-wave (mV)	18.2 ± 2.0	18.6 ± 4.2
VL EMG/M (%)	14.0 ± 5.1	10.0 ± 3.4
VM EMG/M (%)	12.6 ± 5.0	10.3 ± 3.3
VL V/M (%)	21.1 ± 14.4	22.9 ± 8.7
VM V/M (%)	28.7 ± 17.3	21.6 ± 15.1
Q._pot.tw.max_	62.8 ± 6.6	59.7 ± 10.6
Time to reach VOL–2 (s)	211.4 ± 38.9	207 ± 56.8
Time to exhaustion, final contraction (s)	46.1 ± 10.1***	10.8 ± 3.5

***is p<0.001 between protocols.

MVT, maximal voluntary torque; VA, voluntary activation; VL, vastus lateralis; VM, vastus medialis; M-wave, muscle compound action potential; EMG, electromyogram; V/M, V-wave amplitude normalized to M-wave; Q._pot.tw.max,_ quadriceps potentiated twitch maximum. Data are mean ± SD.

### Torque recordings

Participants were required to sit upright in the dynamometer with the hip and knee flexed to 90°. The centre of rotation of the dynamometer lever arm was aligned with the femoral condyle of the left knee. All testing and exercise was performed on the left leg. All participants were right leg dominant. Pilot work in our laboratory has found no consistent pattern related to dominance for between-leg strength differences in trained men. In our laboratory the left leg orientation of the dynamometer was more suitable for display of the LCD monitor for consistent monitoring of torque output during the exercise protocol. The participant was firmly strapped to the seat across the torso, hips, and thigh. The lever arm of the dynamometer was firmly strapped to the lower leg approximately 2cm superior to the lateral malleolus. All torque signals were continuously sampled at 2000Hz, and a 10Hz digital low-pass filtered was applied (Powerlab, ADI Instruments, Sydney, Australia). For measurement of MVT (Nm), participants were required to push as fast and forcefully as possible, and maintain the contraction for 3 to 5s.

### Electromyography

Quadriceps electromyograms (EMG) were recorded from the left vastus lateralis (VL) and vastus medialis (VM) using pairs of Ag/AgCl surface electrodes (Maxsensor, Medimax Global, Australia). VL and VM electrodes (10mm diameter, 10mm inter-electrode distance) were applied after careful skin preparation including removal of excess hair, abrasion with fine sandpaper and cleaning the area with isopropyl alcohol swabs. VL electrodes were placed on the belly of the muscle, with the distal electrode approximately 12-15cm superior to the patella depending on individual differences in muscle shape and tendon length. The distal VM electrode was placed 3-4cm superior to the patella on the muscle. Both pairs of electrodes were angled to approximate the direction of the muscle fibers. Permanent marker was used to maintain electrode positioning between sessions, and placement distances relative to the patella were recorded for each participant. A ground electrode (20mm contact diameter) was applied to the right patella. To minimize movement artefacts, cables were taped down onto the dynamometer chair during all testing. EMG signals were recorded using the ML138 BioAmp (common mode rejection ratio > 85 dB at 50 Hz, input impedance 200MΩ) with 16-bit analog-to-digital conversion, sampled at 4,000 Hz (ADI instruments, Sydney, Australia). Raw signals were filtered with a fourth-order Bessel filter between 20 and 500 Hz. Root-mean-square (RMS) calculations were applied to smooth each muscle recording, using a 100ms moving window.

### Femoral nerve stimulation

The knee extensors were stimulated by a single supramaximal electrical pulse applied to the femoral nerve (0.2ms square wave pulse duration) delivered at 400V using a constant current stimulator (Digitimer DS7AH, Welwyn Garden City, UK). A 20mm diameter Ag/AgCl surface electrode was positioned over the nerve in the femoral triangle, and the anode (20mm, Ag/AgCl electrode) was placed midway between the greater trochanter and the iliac crest. The precise location of the cathode was identified at rest by moving a ball probe and applying low intensity stimulation (40mA) to pre-marked sites within the femoral triangle, with optimal location decided based on the highest evoked M-waves in both VL and VM. The level of stimulation to use during testing was determined by gradually increasing the current until a plateau in knee extensor twitch torque and maximal M-waves (M_max_) for VL and VM was reached (stimulation intensity range, 120-220mA). This intensity was then used to establish the supra-maximal stimulation intensity (130%) that was applied throughout the experimental session. For the interpolated twitch, stimulation was applied on the MVT trial when torque had reached a visible plateau after 1-2s. One twitch was evoked 3-4s after the MVT was stopped and the participant was relaxed to elicit Q._pot.tw_. To examine central motor output during each protocol, stimulation was applied after the first 5s of contraction. In the final contraction to exhaustion, stimulation was applied after the first 5s, and thereafter every 5s until exhaustion.

### Data processing

Contraction onset (for all torque and EMG signals) was identified using the computer-based integrated profile method (MATLAB, The Mathworks, USA), which compares a time normalized function to a normalized function of the integrated signal [20,26]. The point in time where the two normalized functions are the greatest distance apart is identified as signal onset, as this point indicates where the signal of interest begins to rapidly increase with respect to time.

Torque recordings were used to analyse, 1) the maximal voluntary torque recorded during the contraction excluding the point of stimulation (MVT, Nm), 2) rate of torque development (RTD) was calculated as the average slope of the torque-time curve (Δtorque/Δtime) during the time periods 0-25ms, 0-50ms, 0-75ms and 0-100ms post contraction onset, 3) maximum RTD (RTDmax) was calculated as the greatest average 10ms slope throughout the first 100ms of the contraction.

EMG recordings were used to analyse the following variables from each MVT; 1) the electrically evoked muscle compound action potential (M-wave), calculated from the peak-to-peak amplitude of the VL and VM EMG raw signal elicited during contraction, 2) the maximal amplitude of the VL and VM EMG signal during MVTs based on processing the greatest average 250ms root-mean-square (RMS) value, 3) the rate of EMG rise for VL and VM calculated from the average slope of the EMG-time curve during the time periods 0-25ms, 0-50ms, 0-75ms and 0-100ms post contraction onset. In addition EMG was analyzed during the 80% and 40% contractions by processing the average RMS activity in 2s epochs at the start of both contractions, at the end of VOL–1 and VOL–2, and at the end of the final contraction to exhaustion. All EMG variables during maximal contractions were normalized to the respective M-waves elicited during each contraction for data analysis (EMG/M, %).

EMG values at the start and end of the 80% and 40% contractions were normalized to the subsequent M-wave measured during the MVT. M-waves did not change throughout the protocol supporting this normalization choice.

**Fig 2:**
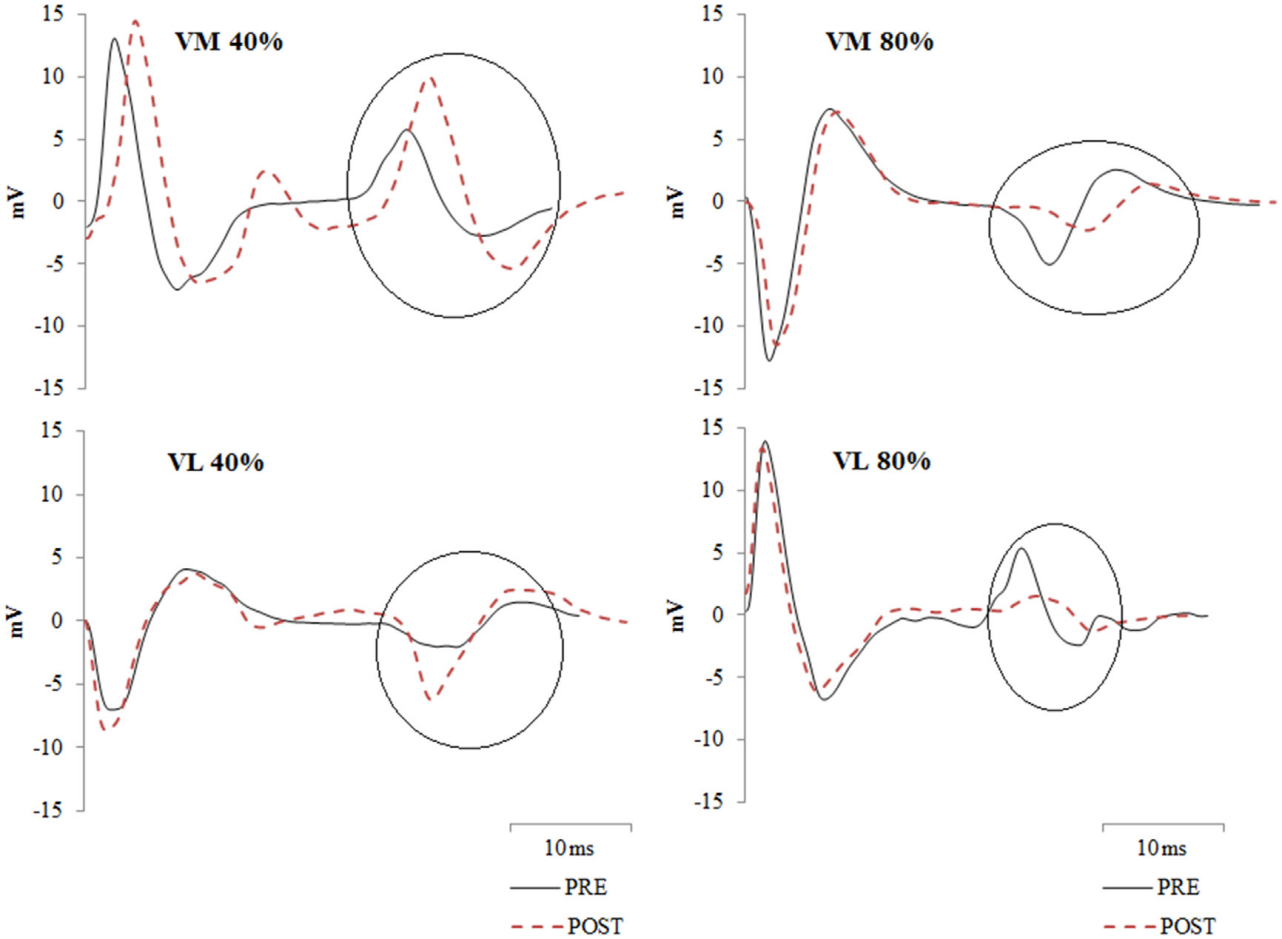
EMG recordings showing the V-waves from a representative participant from vastus medialis (VM) and vastus lateralis (VL) measured pre and post the exercise protocols (40% and 80%). V-waves are circled on the figures. Note the reductions in V-wave amplitude following the 80% protocol.

V-waves are the first evoked potential observed after the M-wave in the EMG recordings. For VL and VM this typically is observed 25 to 30ms post stimulation (Fig 2). The peak-to-peak amplitude of the V-wave was recorded and expressed relative to the M-wave evoked during the same contraction (V/M, %).

Voluntary activation (VA) was estimated using the superimposed twitch technique [16] according to the following formula [27]: VA (%) = 100 –(D * (T_sup_ / MVT) / Q._pot.tw.max_) * 100, where D is the difference between the torque level just before the superimposed twitch (T_sup_) and the maximum torque recorded during the twitch, MVT is maximal voluntary torque during the entire contraction (not including the twitch response), and Q._pot.tw.max_ is the maximal amplitude of the resting potentiated twitch. We also used the above formula to estimate VA from the twitches elicited during the sub-maximal contractions (VA_sub_). Superimposed twitches elicited at the start of each contraction were expressed relative to baseline Q._pot.tw_, whereas the last twitch elicited during the final contraction was expressed relative to the Q._pot.tw_ elicited after the final MVT. For the VA_sub_ formula, MVT was replaced by the average torque (AT, Nm) measured in the 1s period prior to stimulation. Thus VA_sub_ (%) = 100 –(D * (T_sup_ / AT) / Q._pot.tw.max_) * 100.

In addition to the maximal amplitude, the following variables were calculated from Q._pot.tw_: 1) the time to peak twitch (TPT), 2) the ½ relaxation time (½ RT), calculated as the time from the peak amplitude until 50% of the maximal amplitude had been reached, 3) average slope of the torque-time curve during the time periods 0-25ms, 0-50ms, and 0-75ms post onset of the twitch, and 4) maximal rate of twitch development based on the greatest average 10ms slope throughout the twitch.

### Statistical analysis

Apart from baseline MVT (determined from trial with highest value), baseline values for all dependent variables were determined from the average of the two MVT trials performed with femoral nerve stimulation. Data were normally distributed as assessed from inspection of kurtosis and skewness values, in addition to Kolmogorov-Smirnov normality testing. Repeated-measures analysis of variance (ANOVA) procedures were used to examine changes in dependent variables between each protocol (40%, 80%) over time (VOL–1, VOL–2, FINAL). For VA_sub_ ANOVA procedures compared changes between protocol over two levels of time only (5s into first contraction, last twitch during final contraction). Mauchly’s test was used to assess for sphericity and in any cases of violation the Greenhouse-Geisser epsilon correction was used to adjust the degrees of freedom. When a significant f-value was observed in the ANOVA, post-hoc tests with Bonferonni’s correction were used to identify differences. For one participant no V-waves could be detected therefore their data could not be used in V-wave analyses. Descriptive data are mean ± SD. Statistical significance was defined as p ≤ 0.05.

## Results

### Maximal torque and rate of torque development

**Fig 3:**
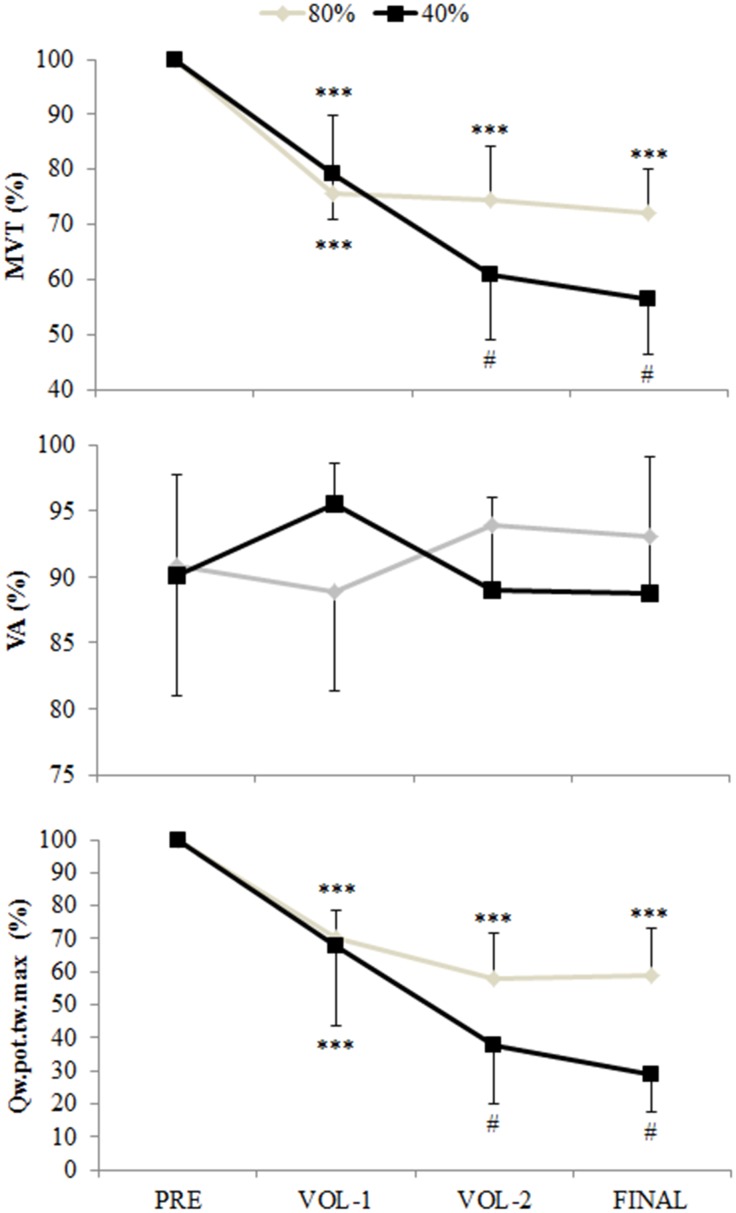
Maximal voluntary torque (MVT), voluntary activation (VA, %), and quadriceps potentiated twitch torque (Q._pot.tw.max_) measured during the 40% and 80% protocols. MVT and Q._pot.tw.max_ are normalized to pre-exercise values. Main effect of time on MVT and Q._pot.tw.max._ *** is p<0.001 from pre-exercise; # indicates interaction between time and protocol, with values reduced from VOL–1 and different from 80% protocol (p<0.01). Data are mean and SD.

**Fig 4:**
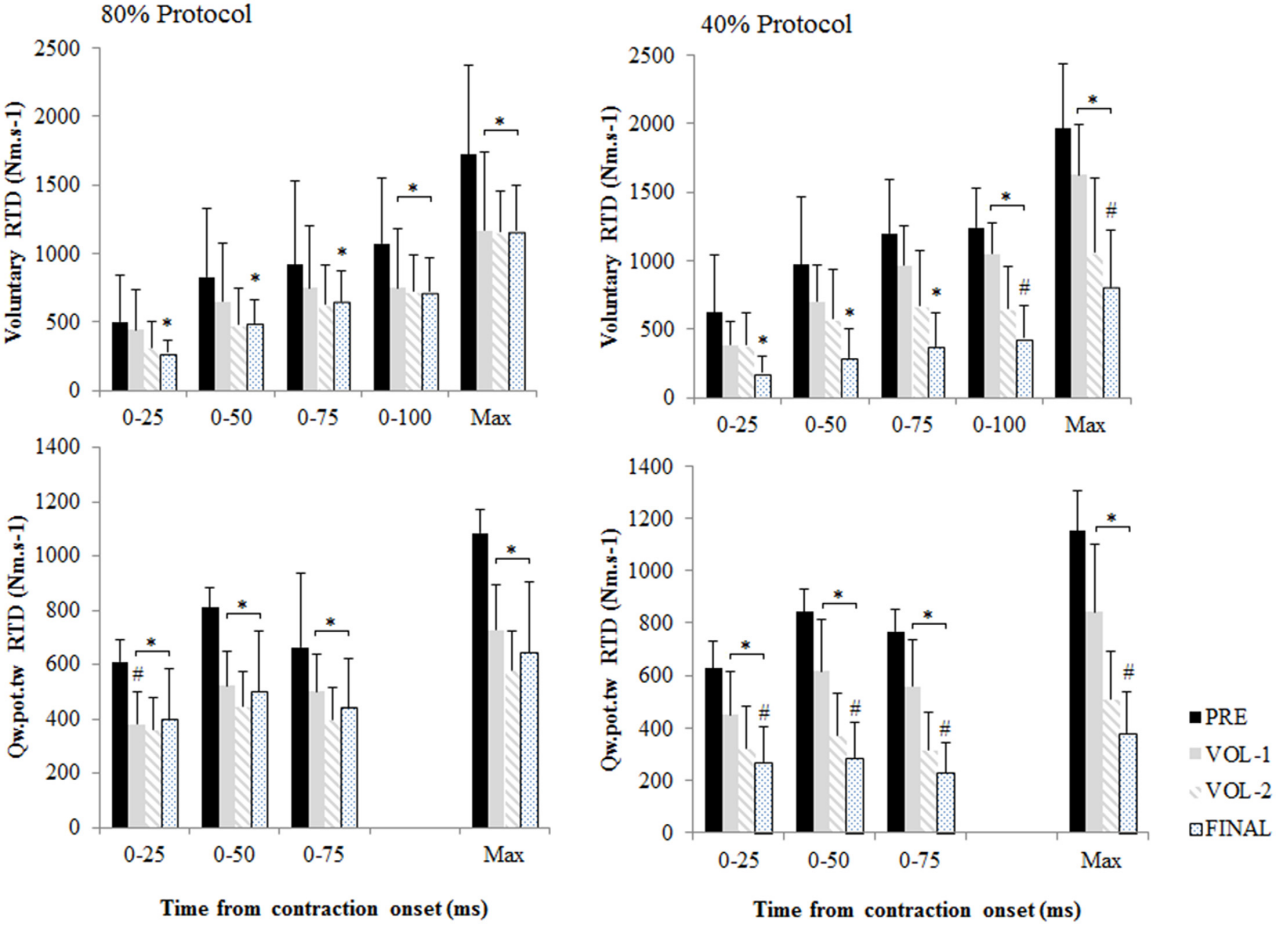
Voluntary rate of torque development (RTD) and rate of torque development of the quadriceps potentiated twitch (Q._pot.tw_) from contraction (or twitch) onset. Q._pot.tw_ was analyzed up to 75ms post onset as analysis up to 100ms would include the relaxation phase of the twitch. * is p<0.01 from pre-exercise. # is p<0.05 between protocols, indicating where interaction effects between time and protocol were observed. Data are mean and SD.

There was a significant interaction between time and protocol for MVT (Fig 3; p = 0.001). MVT for both protocols was similarly reduced from pre-exercise by 21.4 ± 10.2% at VOL–1 (p<0.001). During the 40% protocol MVT at VOL–2 was reduced from VOL–1 by a further 23.5 ± 10.9% (p<0.001), but no further reductions were observed during the 80% protocol. An interaction between time and protocol was observed for RTD 0-100ms (p = 0.003) and RTDmax (p = 0.007). RTD 0-100ms and RTDmax were similarly reduced from pre-exercise at VOL–1 by 19.3 ± 24.7% and 21.3 ± 25.0% respectively (p<0.05). Further reductions were only observed during the 40% protocol, with RTD 0-100ms and RTDmax reduced by 64.1 ± 20.0% and 59.1 ± 16.7% respectively from pre-exercise after the final contraction (p<0.001). Main effects of time were observed for RTD in time intervals of 0–25, 0–50, and 0-75ms post-contraction onset (Fig 4; p<0.003). RTD 0–25, 0–50, and 0-75ms were reduced from pre-exercise after the final contraction by 36.2 ± 57.7%, 39.9 ± 51.4%, and 43.9 ± 40.9% respectively (p<0.05).

### Peripheral muscle fatigue

No changes were observed for VM and VL M-waves. An interaction between time and protocol was observed for the rate of rise in the Q._pot.tw_ in all time intervals from twitch onset (Fig 4; p<0.001), the maximal rate of rise in Q._pot.tw_ (Fig 4; p<0.001), and Q._pot.tw.max_ (Fig 3; p<0.01). For Q._pot.tw_ 0-25ms the 37.6 ± 18.1% reduction observed at VOL–1 during the 80% protocol was greater than the 28.5 ± 18.9% reduction observed during the 40% protocol (p = 0.039). Q._pot.tw_ 0-25ms in the 40% protocol continued to reduce to the end of the final contraction by 57.5 ± 18.6% from pre-exercise (p<0.001). For Q._pot.tw_ 0-50ms, 0-75ms, maximal Q._pot.tw_ rate of rise, and Q._pot.tw.max_, similar reductions from pre-exercise of between 27.3 to 35.5% were observed for both protocols after VOL–1 (p<0.001). During the 40% protocol ongoing reductions were observed for all variables, with values reduced from pre-exercise after the final contraction between 66.2 to 70.8% (p<0.001). After the final contraction, values for Q._pot.tw_ 0-50ms, 0-75ms, maximal Q._pot.tw_ rate of rise, and Q._pot.tw.max_ were lower in the 40% compared to the 80% protocol (p<0.01).

For time to peak twitch, no time, protocol, or interaction effects were observed in the analyses (pre-exercise, 88.0 ± 6.6ms; final, 83.7 ± 15.2ms). For ½ relaxation time (½ RT) an interaction between time and protocol was observed (p = 0.008). Pre-exercise ½ RT were 69.4 ± 35.9ms and 72.9 ± 31.5ms for the 40% and 80% protocols respectively. Similar increases in ½ RT were observed for both protocols after VOL–1 and VOL–2 of 34.9 ± 28.8% and 77.3 ± 59.8% respectively (p<0.01). After the final contraction ½ RT times recorded for the 80% protocol (85.7 ± 15.5ms) were lower than the 40% protocol (141.7 ± 55.6ms; p = 0.045), although no further changes from VOL–2 were observed.

### Central motor output

**Fig 5:**
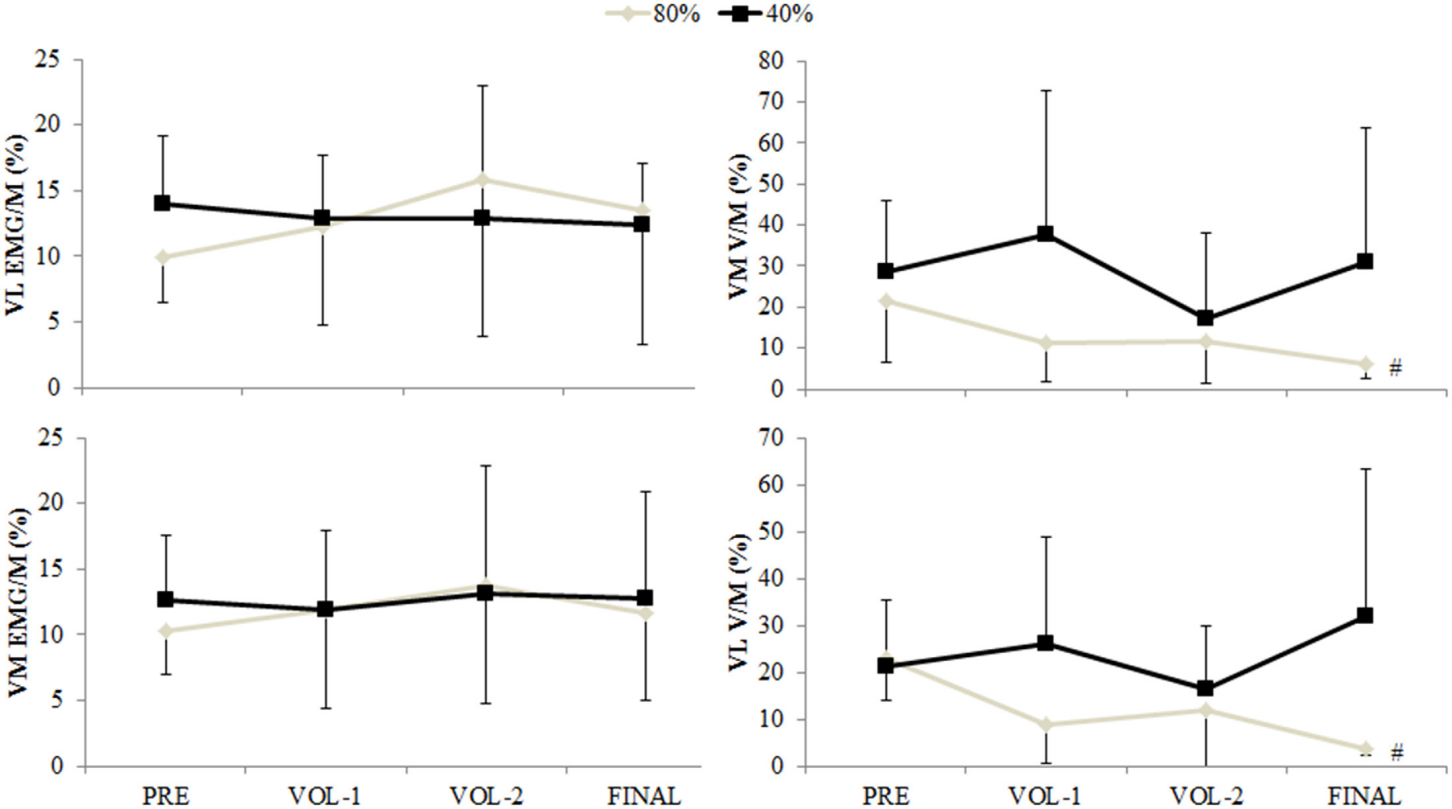
Vastus lateralis (VL) and vastus medialis (VM) maximal EMG and V-waves (normalized to M-waves; EMG/M, V/M) for the 40% and 80% protocols. # indicates interaction between time and protocol, with values reduced from pre-exercise and lower than the 40% protocol (p<0.05). Data are mean and SD.

**Fig 6:**
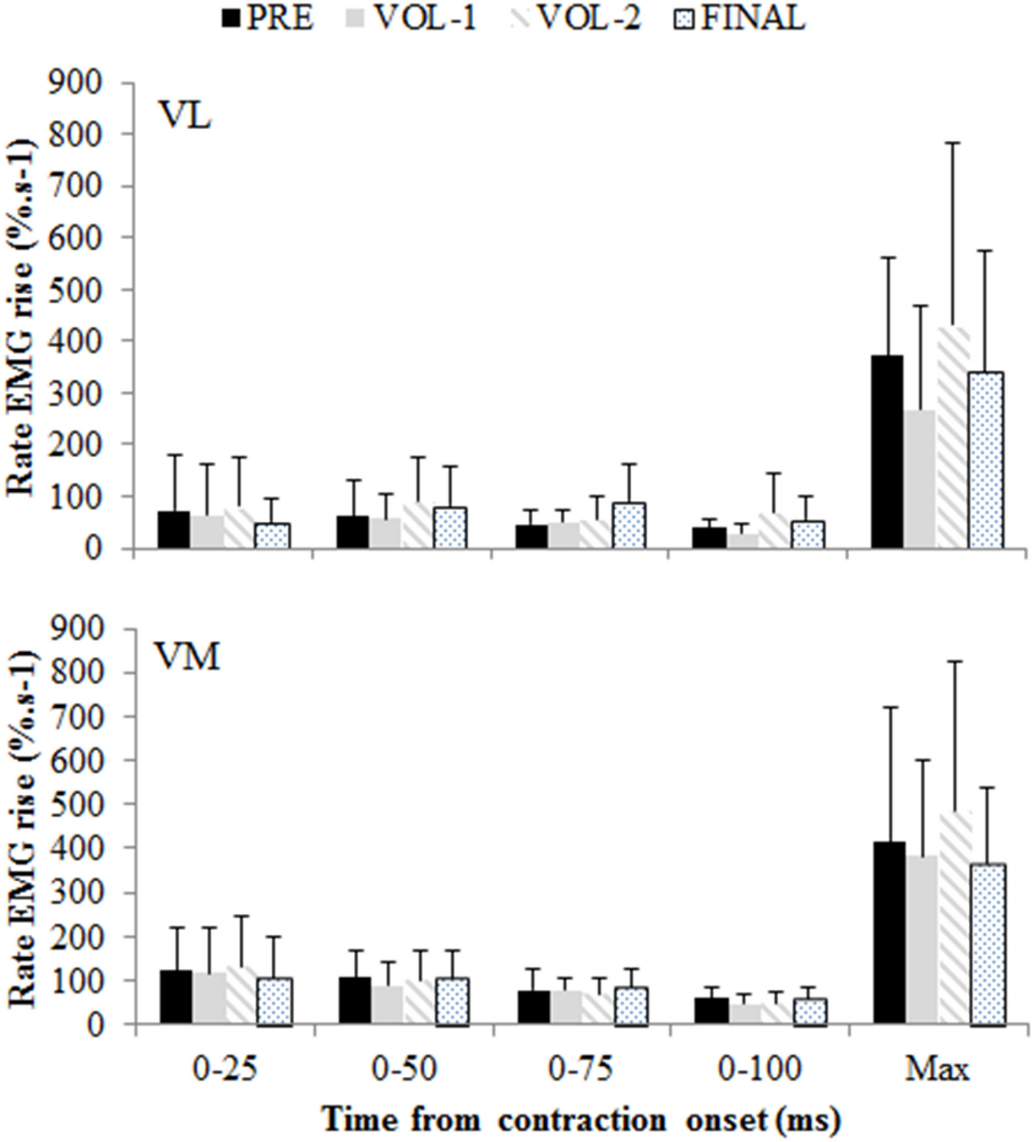
Rate of rise in vastus lateralis (VL) and vastus medialis (VM) electromyograms in time intervals from contraction onset. Pooled results from both protocols are displayed. No time, protocol, or interaction effects were observed. Data are mean and SD.

No main effects of time, or interaction between time and protocol, were observed for VA (Fig 3), EMG/M (Fig 5), and rate of rise in EMG (Fig 6) variables measured during the maximal contractions. An interaction between time and protocol was observed for reductions in VM and VL V-waves (Figs 2 and 5; p<0.05). No changes were observed for V-waves during the 40% protocol. V-waves during the 80% protocol were reduced from pre-exercise after the final contraction for VM and VL by 56.8 ± 35.8% and 53.6 ± 37.4% respectively (p<0.05).

Main effects of time and protocol were observed for VL and VM EMG/M values recorded during the 40% and 80% exercise protocols (Table 2). EMG/M values were higher during the 80% protocol for both muscles (p<0.05). For VM, EMG/M was increased 92.7 ± 94.0% from the start of exercise at VOL–2 (p = 0.004), and for VL EMG/M a 85.5 ± 105.5% increase was observed (p = 0.002).

**Table 2:** Electromyograms (EMG normalized to M-waves; EMG/M, %) recorded from vastus lateralis (VL) and vastus medialis (VM) during the contractions performed at 80% and 40% maximal voluntary torque. A main effect of protocol was observed for VL and VM (p<0.05).

	Start	End VOL–1	End VOL–2	Final
VL (EMG/M, %)				
80% protocol	3.39 ± 0.93	5.68 ± 3.01	6.64 ± 4.78**	6.01 ± 3.56**
40% protocol	2.39 ± 1.25	2.79 ± 1.55	3.99 ± 2.47**	4.00 ± 1.42**
VM (EMG/M, %)				
80% protocol	3.46 ± 0.80	5.67 ± 2.40	6.66 ± 4.42**	5.47 ± 2.52**
40% protocol	1.63 ± 0.67	2.01 ± 0.90	3.13 ± 1.71**	3.56 ± 1.85**

** increased from the start of the protocol (p<0.01). Data are mean ± SD.

VA_sub_ was 91.2 ± 6.2% and 67.1 ± 6.1% at the start of the 80% and 40% protocols respectively (p<0.001). An interaction between time and protocol was observed (p<0.001). At the end of the final 80% contraction VA_sub_ was increased to 94.9 ± 4.7% (p = 0.005), but a greater increase was observed during the 40% contraction where VA_sub_ increased to 88.9 ± 9.6% (p<0.001). VA_sub_ was not different between protocols at the end of the final contraction.

## Discussion

Our results provide unique insight into the contribution of peripheral and central factors to fatigue during single joint lower limb exercise performed at high and low contraction intensities in resistance trained men. The main findings of this study were, 1) maximal central motor output was well preserved in the resistance trained men during both protocols despite worsening peripheral fatigue, 2) reductions in maximal voluntary torque, rate of torque development, and measures of peripheral fatigue were greater in the 40% protocol, and 3) central motor output during the exercise tasks was upregulated, with greater increases observed during the 40% protocol despite worsening peripheral fatigue.

This study supports and extends recent observations that the central nervous system can tolerate different levels of peripheral fatigue during single joint exercise [13,14]. We found no reductions in central motor output during maximal contractions and increased central motor output during the exercise task, particularly during the 40% protocol, despite worsening peripheral fatigue. Maintained central motor output during the maximal contractions contrasts previous work where declines in voluntary activation were observed following fatiguing knee extensor exercise [13,14]. We believe this is best explained by our use of resistance trained compared to recreationally active men [13,14], and suggests that the chronic neural adaptation in this participant group [7–12] may improve the tolerance of the nervous system to varying levels of peripheral fatigue. Indeed, the peripheral fatigue we measured in this study (e.g. between 40 to 70% reduction in Q._pot.tw_) exceeded recent studies on recreationally active individuals reporting a 30 to 40% reduction in Q._pot.tw_ using isolated knee extension exercise [13,14], but unlike these studies we did not observe reductions in maximal central motor output.

In contrast to our hypothesis the overall pattern of results suggests greater disruption of the rate and amplitude characteristics of the Q._pot.tw_ during the 40% protocol. The overall time under tension and volume requirements of the 40% protocol appears to have facilitated progressively greater recruitment of the quadriceps femoris motor unit pool, probably achieved through recruitment of higher threshold motor units and their respective fibers, thus worsening the manifestations of peripheral fatigue. At the first matched volume point (VOL–1, 30s at 80% and 60s at 40%) reductions in both the size and shape of the Q._pot.tw_ were observed for both protocols, although the reduction of Q._pot.tw_ 0-25ms was greater for the 80% protocol. This initial finding provides some support for our hypothesis since preferential fatigue of type II motor units should have affected the rate measures of the Q._pot.tw._ However, after the final contraction to exhaustion both the rate and amplitude measures of the Q._pot.tw_ were reduced to a greater extent after the 40% protocol. Of interest was the time to peak twitch was unchanged in either protocol. This suggests that the temporal characteristics of twitch development were unaffected, and that declines in rate were associated with similar mechanisms that reduced the maximal amplitude of the Q._pot.tw_ (e.g. metabolic perturbation, impaired calcium kinetics and cross-bridge cycling)[1].

Our conclusion of increased central motor output during the 40% protocol was derived from the increased voluntary activation observed in this trial, as estimated from the interpolated twitch applied during the exercise protocols (VA_sub_). This is a relatively novel application of the interpolated twitch technique, which is typically used during maximal contractions. We believe this method has greater validity for estimating central motor output to a muscle group during an exercise task as compared to surface EMG. Limitations to the interpretation of the surface EMG signal, particularly with respect to EMG recordings during fatiguing contractions, have been well documented [28,29]. Specifically, surface EMG signal lacks sensitivity to detect changes in the number and discharge rate of active motor units that would indicate increased central motor output [25,30]. In this study EMG amplitudes (EMG/M, %) increased during both exercise protocols, although activity was always higher for the 80% protocol which may be interpreted to suggest greater central motor output compared to the 40% contraction. Similar to the EMG comparison measured at the start of the exercise protocols, sub-maximal voluntary activation was different at the start of the 80 and 40% protocols with VA_sub_ of 91.2 ± 6.2% and 67.1 ± 6.1% respectively. This observation from VA_sub_ supports the premise of Henneman’s size principle in that a progressively greater recruitment of the available motor unit pool is required to facilitate a higher intensity contraction. EMG amplitudes increased during both protocols, but no interaction effects were observed suggesting central motor output changed similarly but was never equivalent. However the aforementioned limitations about surface EMG confounds any conclusion beyond a general trend in both protocols for increased central motor output. In contrast to the surface EMG results, VA_sub_ increased in the 40% protocol to a greater extent than the 80% protocol, and there were no differences between protocols at the end of the final contraction. Indeed VA_sub_ at the end of both contraction protocols was equivalent to VA measured during the maximal voluntary contractions, suggesting that both tasks achieved full voluntary activation individuals were capable of.

The basis of concluding no reductions or between-protocol differences in central motor output during the maximal voluntary contractions was from no change in the measures of voluntary activation (Fig 3), maximal surface EMG amplitudes (Fig 5), and the rate of rise in the surface EMG signals (Fig 6). However the final measure thought to be indicative of central motor output, the normalized V-wave (V/M, %), indicated a between protocol difference with greater reductions observed during the 80% protocol (Figs 2 and 5). By itself this finding could indicate a protocol specific reduction in central motor output, but the lack of substantiation from other measures refutes this premise. It must be considered that the amplitude of the V-wave is also mediated by physiological factors similar to that which influence the H-reflex. Specifically, this includes pre- and post-synaptic influences of the Ia afferent onto the α-motoneuron output [8,19], descending drive as well as postsynaptic excitatory and inhibitory spinal inputs [31]. Changes in pre-synaptic inhibition during fatiguing exercise have been reported. Nordlund and colleagues used a paired pulse technique to evoke concurrent H-reflexes in the soleus during 90 maximal plantarflexion efforts to explore pre-synaptic inhibition of the Ia afferent [32]. While the authors concluded that decreased pre-synaptic inhibition occurred during the fatiguing contractions, they could not specify whether this was owing to decreased GABA mediated inhibition of the Ia afferent or homosynaptic post-activation depression [32]. Another study utilized the paired pulse technique to examine pre-synaptic modulation of Ia afferents for the extensor carpi radialis [33]. The authors observed longer endurance time during a constant 20% force task and decreased presynaptic inhibition, whereas endurance time was less but presynaptic inhibition tended to increase for a position task where a 20% load was maintained at a constant angular position [33]. It was suggested that depression of Ia input onto the α-motoneuron was a strategy to maintain the constant limb position requirement of the task. Combined, these studies suggest that Ia presynaptic inhibition is modulated differently depending on task requirements. The results of the current study extends this summary to suggest that Ia afferent presynaptic inhibition is also modulated according to the intensity of the fatiguing contractions, with greater inhibition elicited from the 80% protocol. However we are unable to discriminate the mechanisms underpinning this change with the stimulation method used in this study.

Finally there are some limitations within our study to address. First, we only placed surface electrodes on the vastus lateralis and medialis. Therefore we cannot be sure that other muscles of the quadriceps muscle group (i.e. rectus femoris and vastus intermedius) would have exhibited the similar changes, or lack thereof, observed in this study. Moreover these findings are only generalizable to lower limb single-joint exercise. Different responses may be observed in single joint exercises performed in the upper limb, where a muscle such as the triceps brachii typically exhibits a much higher proportion of type II muscle fibers compared to the quadriceps or calf muscles [23]. Of interest with regards to the design and performance of the exercise protocols, not all participants could complete the exercise protocols up to the accumulation of the second amount of volume without requiring rest (60s total contraction time at 80%, 120s at 40%). However the total time required, including rest, to reach this volume of work (VOL–2) was similar between the exercise protocols. Therefore the ‘density’ of the 40% and 80% exercise protocols was comparable at the VOL–2 measurement point, and we believe a valid comparison of an equitable volume of exercise. The use of a single pulse did not allow examination of mechanistic factors that may explain why the V-waves declined in the 80% protocol. Future research could utilize paired-pulse stimulation techniques to probe pre- and post-synaptic inhibition mechanisms. We did not use a healthy, untrained control group to compare manifestations of fatigue at the different intensities. While a control group could provide greater delineation for the changes, or lack thereof, observed in this study, we believed that there has been ample research provided in the literature with regards to declines in central motor output and muscle twitch responses in healthy untrained populations to contrast our data. Our laboratory has recently published data demonstrating reduced central motor output of the calf and quadriceps muscle groups following exhaustive single joint exercise in untrained participants, commensurate with the typical findings observed in this population [20,34]. Also, we cannot generalize our findings to the expected outcomes and differences from training programs based on the 40% and 80% protocols. Some data suggests that low intensity exhaustive exercise programs that accrue greater training volume elicit similar isometric strength gains and hypertrophy outcomes compared to high intensity programs [35]. However this data is from untrained participants, and cannot be generalized to a resistance trained population. At present there is no data adequately comparing lifting intensities for strength and hypertrophy outcomes in trained populations. Performing such a training study may be difficult for participant recruitment, as the use of exhaustive exercise at 40% intensity would contradict existing dogma for how strength programs in trained individuals are prescribed. However, such data is necessary to examine the relevance of the acute fatigue responses observed in this study with training outcomes.

## Conclusion

The present findings are the first to demonstrate that maximal central motor output in resistance trained men is well preserved despite varying levels of peripheral muscle fatigue. Moreover, central motor output during the 40% contraction protocol was upregulated to levels commensurate with the 80% protocol that, combined with the greater time of contraction, elicited greater levels of peripheral fatigue. Greater reductions in V-waves during the 80% protocol suggest intensity dependent modulation of Ia afferent input to the α-motoneuron. Finally, it is not possible to state whether the acute changes observed in this study would be indicative of any future resistance training adaptation to the respective protocols.
